# De novo* ATP1A2* variants in two Chinese children with alternating hemiplegia of childhood upgraded the gene–disease relationship and variant classification: a case report

**DOI:** 10.1186/s12920-021-00947-6

**Published:** 2021-04-01

**Authors:** Danping Huang, Min Liu, Hongying Wang, Bingbing Zhang, Dongjing Zhao, Weihao Ling, Manli Wang, Jun Feng, Yiping Shen, Xuqin Chen

**Affiliations:** 1 Department of Neurology, Children’s Hospital of Soochow University, No. 92 Zhongnan Street, Soochow, 215000 Jiangsu China; 2 Department of Laboratory, Children’s Hospital of Soochow University, Soochow, Jiangsu China; 3 Genetic and Metabolic Central Laboratory, Birth Defect Prevention Research Institute, Maternal and Child Health Hospital, Children’s Hospital of Guangxi Zhuang Autonomous Region, No. 225 Xinyang Street, Nanning, 530000 Guangxi China; 4 Division of Genetics and Genomics, Boston Children’s Hospital, Boston, MA USA

**Keywords:** Alternating hemiplegia of childhood, *ATP1A2*, De novo, Variant classification

## Abstract

**Background:**

*ATP1A2* gene mutation has been indicated to cause alternating hemiplegia of childhood (AHC); however, limited evidence supports this relationship so far.

**Case presentation:**

We reported two Chinese patients with de novo* ATP1A2* variants (c.970G>A and c.889G>A). Both patients presented with episodes of alternating hemiplegia, seizures and mild developmental delay. Brain magnetic resonance imaging revealed abnormal signals in both patients.

**Conclusions:**

The new genetic evidence we reported here strengthened the gene–disease relationship, and the gene curation level between *ATP1A2* and AHC became “Moderate” following the ClinGen Standard Operation Procedure. Consequently, the two variants can be reclassified as likely pathogenic.

## Background

Alternating hemiplegia of childhood (AHC) is a rare syndrome that is characterized by recurrent and varying degrees of alternating hemiplegia [1], as well as other movement disorders such as dystonic posturing, choreoathetoid movements, and ocular motor abnormalities [2]. Progressive cognitive impairment is also an important feature of this disease [3]. Pathogenic variants in *ATP1A2* are known to cause AHC type 1 (OMIM# 104290), but only a very limited number of cases have been reported [4–6]; thus, the gene (*ATP1A2*)-disease (AHC) relationship is “Limited” according to the ClinGen gene curation protocol (https://clinicalgenome.org/docs/summary-of-updates-to-the-clingen-gene-clinical-validity-curation-sop-version-7/). Pathogenic variants in *ATP1A2* are more commonly associated with familial hemiplegic migraine (FHM) [7]. Here, we report two Chinese boys with de novo mutations in *ATP1A2* who presented with mild growth and developmental delays, seizures, alternating hemiplegia, and abnormal MRI signals. The reporting of these two cases can elevate the level of gene–disease association from “Limited” to “Moderate”, thus further strengthening the causal relationship between *ATP1A2* and AHC.

## Case presentation

We identified two Chinese AHC patients at the Children’s Hospital Affiliated with Soochow University from August 2016 to August 2020. The patients were molecularly diagnosed by exome sequencing, and the diagnosis was confirmed by Sanger sequencing. The study followed the principles of the Declaration of Helsinki and was approved by the Institutional Review Committee of Children’s Hospital Affiliated with Soochow University. Protocol Written informed consent was obtained from the patients’ parents.

Clinical chart review provided information regarding family history, case histories and neurological symptoms and onset. Magnetic resonance imaging (MRI) (3.0T) scans and electroencephalogram (EEG) were performed in both patients.

### Case 1

Case 1 was a 4-year-old boy with febrile seizures, epileptic seizures, and recurrent hemiplegia. He had two episodes of acute hemiplegia within 1 year. Each episode was preceded by mild fever and generalized tonic–clonic seizures lasting less than 5 min followed by several days of hemiparesis. Delayed motor development was exhibited: he was able to hold his neck up at 3 months and to walk unassisted at 15 months. There was no family history of similar symptoms, migraine or seizures.

**Fig. 1:**
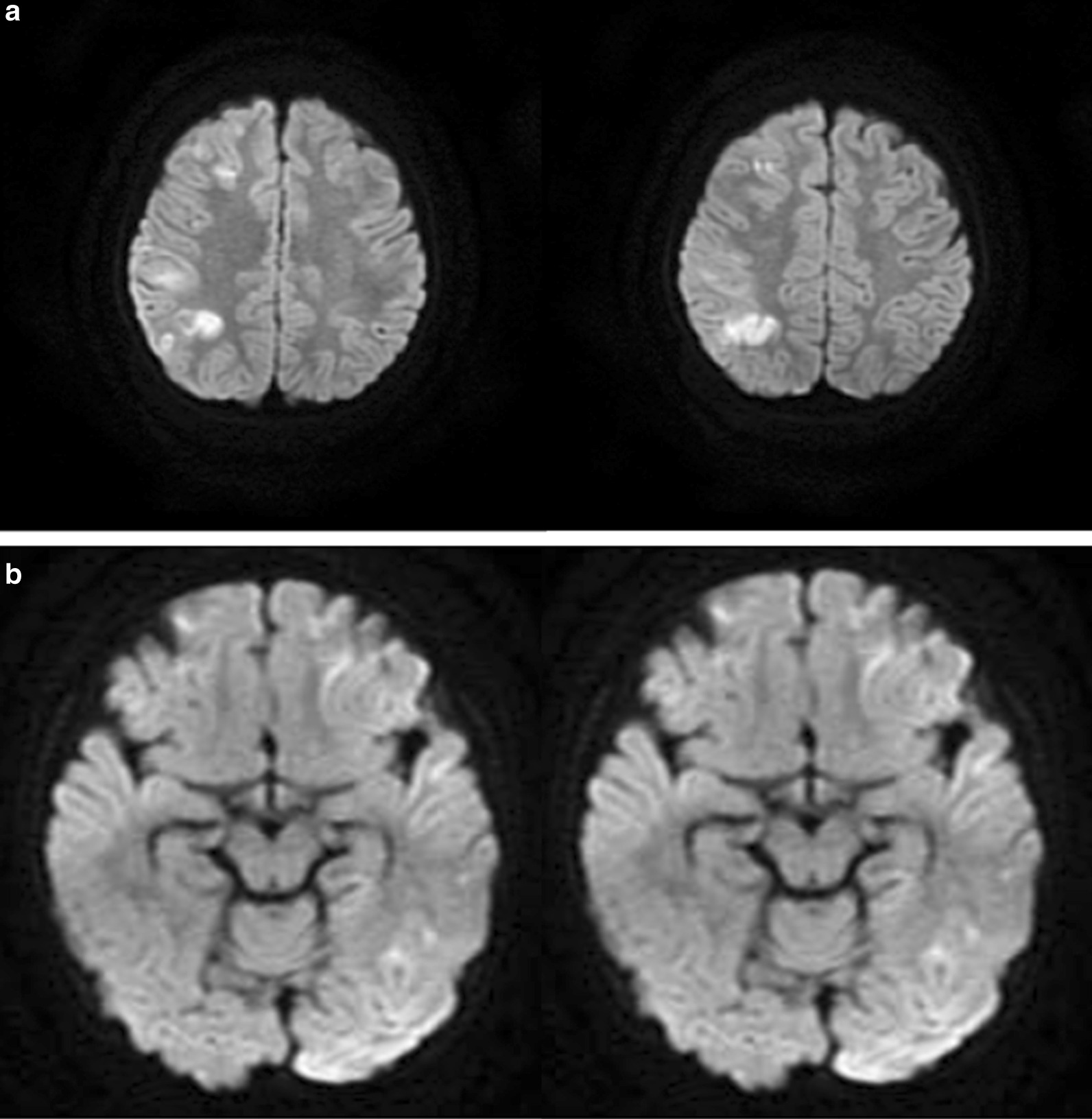
Brain diffusion-weighted magnetic resonance imaging (MRI-DWI) in case 1 (**a**) and case 2 (**b**). **a** Brain MRI findings (axial; diffusion-weighted images) of case 1 (first hemiplegic episode). Cortical restricted diffusion signals were seen predominantly in the right frontal and parietal lobe during the episode of left hemiplegia. **b** Brain MRI findings (axial; diffusion-weighted images) of case 2. Mild hyperintensity was seen in the left hemispheric cortex during the episode of right hemiplegia

**Fig. 2:**
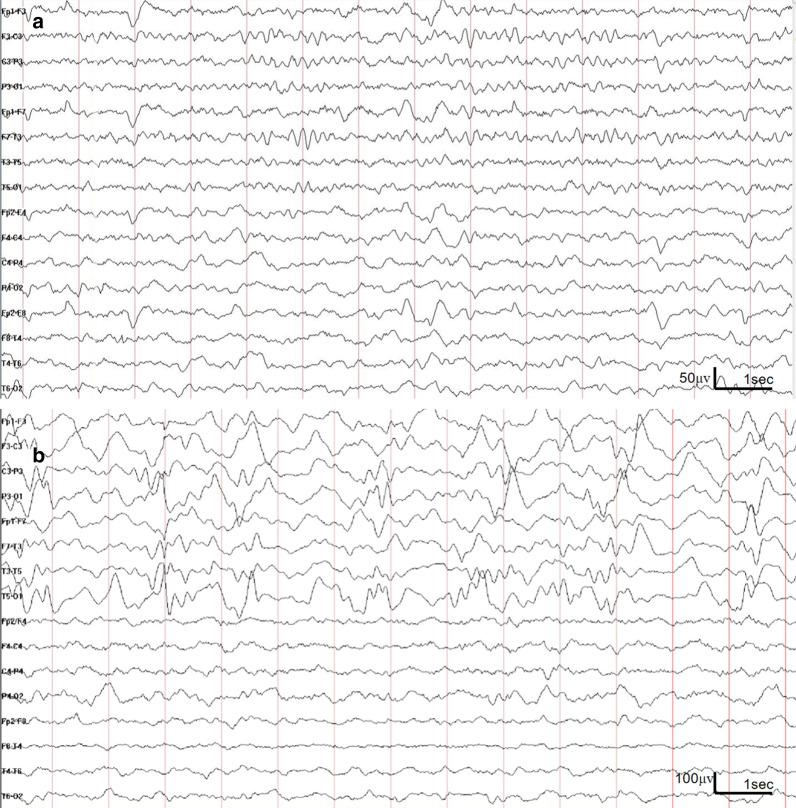
Electroencephalogram (EEG) in case 1. **a** Diffuse slow waves were seen throughout the waking and sleeping phases, especially in the right hemisphere (first hemiplegic episode). **b** Diffuse high-amplitude slow waves were seen throughout the waking and sleeping phases in the left hemisphere (second hemiplegic episode)

Brain diffusion-weighted magnetic resonance imaging (MRI-DWI) showed transient unilateral cortical restricted diffusion signals during a severe episode of hemiplegia (Fig. 1a). EEG showed diffuse slow waves throughout the waking and sleeping phases, especially in one hemisphere (corresponding to the abnormal MRI signal side) (Fig. 2a). His second hemiplegic episode was not accompanied by obvious abnormalities on MRI, but EEG changes (Fig. 2b) were found, leaving him with persistent aphasia for over a year.

### Case 2

Case 2 was a 19-month-old boy from a nonconsanguineous couple who was admitted to our hospital because of fever and right hemiplegia after one febrile convulsion (generalized tonic–clonic seizures and right-sided clonic seizures lasting less than 5 min). Neurological examination indicated right hemiparesis for 2 days but no other neurological signs. He had a similar episode 2 months before admission, which did not draw attention. Brain MRI showed mild DWI hyperintensity involving the left hemispheric cortex (Fig. 1b), while his EEG was normal. He was able to hold his neck up at 3 months, sit without support at 8 months, and walk unassisted at 15 months. He had no positive family history.

The clinical data of the two Chinese patients with *ATP1A2* mutations are summarized in Table 1.

**Table 1:** Clinical data of the two patients with *ATP1A2* mutations

Clinical data	Patient1	Patient2
Sex	Male	Male
Age of onset	4 year	1 year 7 month
Hemiparesis	Yes	Yes
Oculomotor abnormalities (nystagmus, gaze deviation, strabismus)	No	Yes
Disappearance of symptoms with sleep	Yes	Yes
Febrile convulsion	Yes	Yes
Developmental delays	Yes	Yes
Barin MRI	Abnormal	Abnormal
EEG	Abnormal	Normal
Cerebrospinal fluid (CSF)	Normal	Normal
Liquid chromatography-tandem mass spectrometry method (LC/MS)	Normal	Normal
Genotype	De novo missense variant c.970G>A, p.G324S	De novo missense variant c.889G>A, p.A297T

## Results

Variants in *ATP1A2* (NM_000702) were identified by whole-exome sequencing: c.970G>A: p.Gly324Ser [Chr1(GRCh37):g.160097563G>A] in case 1 and c.889G>A: p.Ala297Thr [Chr1(GRCh37):g.160097482G>A] in case 2. These variants were confirmed by Sanger sequencing in the patients and their parents and were found to be de novo variants. These two variants are classified as "likely pathogenic" following ACMG guidelines [8]. The variant c.970G>A: p.Gly324Ser of case 1 has not been reported in the literature. The variant c.889G>A: p.Ala297Thr of case 2 was reported [9] to be associated with dystonia but not hemiplegia. Both variants are located in the intracellular region of the Na+/K+-ATPase a2 subunit.

As these two variants have not been reported to be related with AHC in the literature, to facilitate the evaluation of the clinical significance of each variant, we searched the same variant submitted in ClinVar (http://www.ncbi.nlm.nih.gov/clinvar/), which displays supporting data from each submission and determines whether the submitted clinical interpretations are conflicting or concordant [10]. Searching ‘c.970G>A, c.889G>A’ in ClinVar returns one and four submissions, respectively. All these submissions were classified as variants of uncertain significance (VUS) in ClinVar. (Table 2).

**Table 2:** List of search results in ClinVar

Individual	cDNA	Protein	De novo	GnomAD	ACMG scoring	Pathogenicity
Patient 1	c.970G>A	p.G324S	Yes	Not reported	PS2 + PM1 + PM2 + PP3	Likely path
1	c.970G>A	p.G324S	No	Not reported	PM1 + PM2 + PP3	VUS
Patient 2	c.889G>A	p.A297T	Yes	Not reported	PS2 + PM1 + PM2 + PP3	Likely path
1	c.889G>A	p.A297T	No	Not reported	PM1 + PM2 + PP3	VUS
2	c.889G>A	p.A297T	No	Not reported	PM1 + PM2 + PP3	VUS
3	c.889G>A	p.A297T	No	Not reported	PM1 + PM2 + PP3	VUS
4	c.889G>A	p.A297T	No	Not reported	PM1 + PM2 + PP3	VUS

*de novo* parents available to confirm de novo status, *likely path* likely pathogenic, *VUS* variants of uncertain significance. Each pathogenic criterion is weighted as very strong (PVS), strong (PS); moderate (PM), or supporting (PP)

Following these results, case 1 was treated with adenosine disodium triphosphate tablets and oxcarbazepine for his seizures, with no further hemiplegic episodes since the last admission. At his last follow-up, the strength of his limbs had completely returned. Although he still has speech problems, he has made great progress in following his parents' instructions. The hemiparesis of case 2 is basically restored, with no further attacks so far.

## Discussion and conclusions

We report the clinical and genetic data of two Chinese AHC patients with de novo* ATP1A2* variants. Febrile convulsions, AHC, mild developmental delay and MRI abnormalities were present in both patients. One patient had epilepsy with persistent aphasia. Postictal aphasia may be a feature of Todd's paralysis. We describe two children with episodes of hemiplegia after generalized tonic–clonic seizures. However, these seizures stop spontaneously within 5 min. Their alternating attacks and developmental delay support the diagnosis of AHC rather than Todd's paralysis. To our knowledge, although the association of *ATP1A2* variants with epilepsy and hemiplegic attacks has previously been described, phenotypic overlap with aphasia and MRI abnormalities has not yet been reported.

Our patients exhibited the clinical features of AHC. However, our patients are also distinguished from the majority of classic AHC patients by the late age of onset (typically < 18 months), the lack of recurrent dystonia, and autonomic nervous manifestations associated with seizures [11–13]. Abnormal brain MRI is uncommon for AHC but has been reported previously [5]. However, persistent aphasia after hemiplegia has not been reported. Our study shows that the phenotype of patients with *ATP1A2* mutations is highly variable in terms of the diversity of clinical manifestations. Therefore, our patients’ presentations extended the clinical phenotype of Chinese AHC associated with the *ATP1A2* pathogenic variant.

*ATP1A2* was initially identified to be associated with familial hemiplegia migraine (FHM) type 2 [14]. Several studies [4–6] have implicated *ATP1A2* variants as uncommon causes of AHC, and the *ATP1A2* variants are also associated with epilepsy and mental retardation [5]; however, few such cases have been reported, and none have been reported in Chinese patients so far. Based on the published cases, the gene–disease relationship regarding clinical validity is at a limited level per ClinGen gene curation classification (https://clinicalgenome.org/docs/summary-of-updates-to-the-clingen-gene-clinical-validity-curation-sop-version-7/). To reach an affirmatory level, additional cases or functional evidence is needed. In our patients, two novel de novo variants (c.970G>A, c.889G>A) of *ATP1A2* were identified and classified as "likely pathogenic" following ACMG guidelines [8]. These two cases provided sufficient evidence to reclassify the gene–disease relationship as “Moderate” (the total clinical validity score reached 8).

Furthermore, because these two variants have not been reported to be related with AHC in the literature, we searched ClinVar (http://www.ncbi.nlm.nih.gov/clinvar/) to further understand the *ATP1A2* mutations mentioned above. The Variation Report presents all submissions referencing given variants. Interestingly, both of the novel variants have been submitted in ClinVar, while both of the submissions’ variance classifications were VUS. According to the ACMG standard [8], all submissions meet criteria for PM1 (located in a mutational hot spot and/or critical and well-established functional domain without benign variation), PM2 (the variants are not present in population databases) and PP3 (algorithms developed to predict the effect of missense changes on protein structure and function suggest that the variants are likely to be disruptive, but these predictions have not been confirmed by published functional studies and their clinical significance is uncertain). In summary, as the available evidence is currently insufficient to determine the pathogenicity of the two variants in disease, they have been classified as a VUS. Hence, the distinction between a deleterious mutation and a rare polymorphic variant may be difficult in isolated cases when the variant is unknown, except when de novo occurrence can be established [15]. Our patients provide crucial evidence that these two VUS could be upgraded as likely pathogenic. Ultimately, based on our two patients, five other patients carrying either of the two mutations submitted in ClinVar (http://www.ncbi.nlm.nih.gov/clinvar/) all around the world could be diagnosed genetically.

In conclusion, we reported two novel de novo* ATP1A2* variants and additional phenotypes associated with AHC in two Chinese patients. This case series not only further strengthened the causal relationship between *ATP1A2* and AHC but also extended the clinical phenotype of Chinese AHC associated with *ATP1A2* pathogenic variants. More importantly, our study upgraded the variant classification of the two novel *ATP1A2* variants (c.970G>A, c.889G>A). Our study is a good example of a rare case series that helps to interpret genetic tests in molecular diagnosis practice and upgrade the variant classification in ClinVar.

## Data Availability

The datasets generated and/or analysed during the current study are available in the NCBI repository, [SAMN18376542; SAMN18376543; SAMN18376544; SAMN18376545; SAMN18449888; SAMN18449889]. These materials described in the manuscript, including all relevant raw data are available upon request.

## Abbreviations

AHC: Alternating hemiplegia of childhood
VUS: Variants of uncertain significance
FHM: Familial hemiplegic migraine
